# The Influence of Tomographic Corneal Characteristics on Epithelial Thickness Profile

**DOI:** 10.7759/cureus.11731

**Published:** 2020-11-27

**Authors:** Nauman Hashmani, Maria Hashmani, Sharif Hashmani, Kiran Fatima, Neha Farid, Faiza Zakaria, Mujtaba A Qazi

**Affiliations:** 1 Ophthalmology and Visual Sciences, Hashmanis Hospital, Karachi, PAK; 2 Oral and Maxillofacial Surgery, Darul Sehat Hospital, Karachi, PAK; 3 Ophthalmology, Dow Medical College, Dow University of Health Sciences, Karachi, PAK; 4 Ophthalmology, Pepose Vision Institute, St. Louis, USA

**Keywords:** as-oct, oct, cornea, corneal tomography, pentacam, corneal astigmatism, corneal epithelium, optical coherence tomography, corneal topography

## Abstract

Purpose

To understand the influence of tomographic corneal characteristics on the epithelium of normal eyes.

Methods

We scanned a total of 98 eyes of 98 individuals using anterior segment tomography and a spectral-domain optical coherence tomography (OCT) epithelial mapping tool. Only eyes with no previous pathology were included, with a refractive range of +5 diopters (D) to -6 D, intraocular pressure of < 22 mmHg, and no evidence of dry eye (Schirmer’s test 2 value > 5 mm). Corneal curvature metrics were statistically correlated with regional epithelial thickness parameters.

Results

The anterior and posterior corneal surface flat and steep axis, the maximum and minimum curvature, corneal topographic astigmatism, astigmatism polar values, and corneal volume had no statistically significant correlation (p>0.05) with the epithelial thickness. Similarly, anterior corneal surface asphericity had no significant correlation. Posterior surface asphericity had a statistically significant moderate correlation with the epithelium in all areas. Similar results were seen in the multivariate analysis.

Conclusions

None of the front or back surface parameters had any influence on the corneal epithelium except for the posterior surface asphericity. This statistically significant yet clinically insignificant correlation may be enhanced in diseased populations like keratoconus and could indicate epithelial remodeling with early posterior corneal changes.

## Introduction

There has recently been a strong interest in defining the epithelial thickness profile in human corneas. Various studies have mapped epithelial profiles in the normal population [1-3], with the goal of assisting in the detection of corneal pathologies, such as keratoconus [4], and to help understand the mechanism of post-laser-assisted in situ keratomileusis (LASIK) regression [5]. Various instruments like very high-frequency digital ultrasound [6] and optical coherence tomography (OCT) [1] can help us understand this distribution in detail.

We know that the epithelium has an asymmetric distribution and volume across the cornea, both in the vertical and horizontal direction [1]. These thickness maps can help us better define the factors that affect this distribution. For example, keratoconus causes the epithelium to remodel in a characteristic doughnut shape [4].

To our knowledge, no study has observed the effect of quantitative tomographic parameters on epithelial thickness distribution in normal corneas. Therefore, we studied the association of a number of anterior and posterior curvature parameters, such as the astigmatism magnitude and axis and corneal asphericity (Q-value), with the distribution of the epithelial thickness profiles in the central 5 mm.

## Materials and methods

Subjects

This was a cross-sectional study that took place at the Department of Opthalmology and Visual Sciences, Hashmanis Hospital, Karachi, Pakistan. We sought informed consent from every patient included, and the ethics board of the hospital gave approval for the study in accordance with the Declaration of Helsinki.

Tests

Each participant underwent the following tests: slit lamp and dilated fundus examination, auto refractometer (Topcon KR-800, Tokyo, Japan), Snellen chart for visual acuity, manifest refraction, intraocular pressure (IOP) via an air-puff tonometer (Reichert 7CR, Reichert, Inc., Depew, NY), ultrasonic axial length calculation (Wavelight OB-820, WaveLight, Erlangen, Germany), corneal tomography (Pentacam HR; Oculus, Wetzlar, Germany), and an anterior and posterior segment spectral-domain OCT (Optovue, Inc). We scanned eyes during the hours of 8:00 AM to 1:00 PM to account for diurnal variation. Additionally, we randomly picked one eye from each patient to be included in the analysis. Corneal tomography was the first test performed to limit artifacts from ocular examination and additional testing.

**Figure 1:**
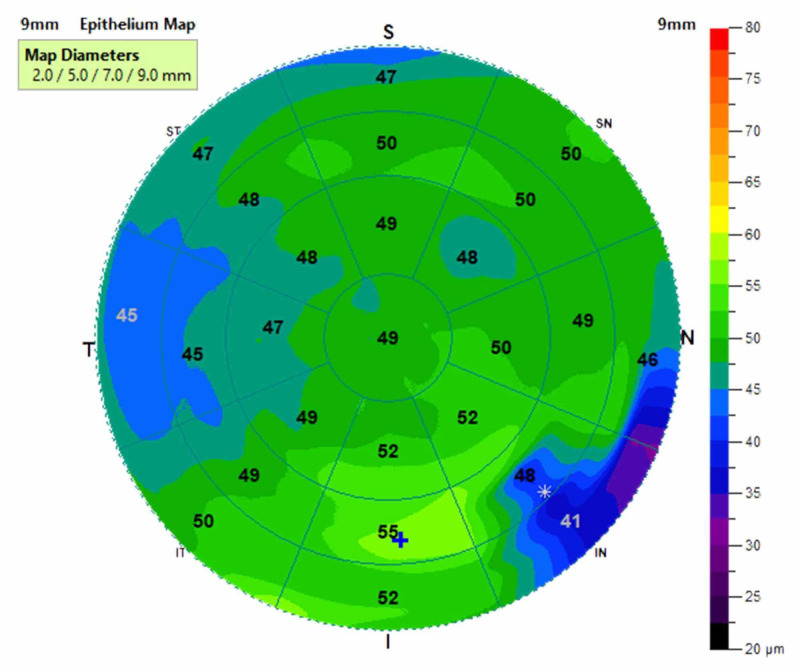
An example of the 9 mm corneal epithelial map Data were analyzed in the central 5 mm zone.

An example of an epithelial thickness profile map is given in Figure 1. The values from the central 5 mm (9 zones) were taken and divided as the figure shows. One value was taken from the center and eight from the paracentral zones. Each zone is an average of the area covered, as shown. The Belin Ambrosio Deviation D value (BAD) value is calculated automatically by the corneal tomographer using five different parameters: anterior corneal elevations, posterior corneal elevations, thickness progression from thinnest point to the peripheral cornea, thinnest point pachymetry, and thinnest point displacement value.

Inclusion/exclusion

We used the following inclusion criteria: refractive error within +5 diopters (D) to -6 D; best-corrected distance visual acuity (BCVA) > 0.8; intensive outpatient treatment (IOP) < 22 mmHg. Patients with a history of cataract, glaucoma, ocular surgery, visual field loss, amblyopia, systemic disease, pregnancy, or those with evidence of dry eye (Schirmer’s test 2 value < 5 mm) were excluded. The tomographic and epithelial scans of every patient were checked for corneal pathologies such as keratoconus by two experienced consultant ophthalmologists; those with evidence of these abnormalities were excluded as well. Lastly, patients with a history of contact lens wear, ocular allergies, or those on topical medications were omitted.

Corneal astigmatism

The value was acquired in a 3 mm zone centered around the pupil. We analyzed these values using two separate methods. In the first, we simply used the magnitude for evaluation. In the second method, we used a vector-based approach outlined by Næser et al. [7], where the astigmatism values were divided into two polar values. The first, KP(45), along the 45-degree meridian, and the second, KP(0), along the zero degrees meridian.

Statistical analysis

All analysis was performed using Statistical Package for the Social Sciences (SPSS v23; SPSS, Inc., Chicago, IL). The Shapiro-Wilk test was used to check for normality. Descriptive statistics were used for the mean and standard deviations. The Pearson correlation was applied for the rest of the variables. Lastly, a multivariate analysis was performed.

## Results

General characteristics

The mean age in our population was 36.8 ± 14 years, which included 33 males and 65 females. The rest of the general characteristics are listed in Table 1.

**Table 1:** General characteristics Abbreviations: SD=Standard deviation; K1=Flat axis; K2=Steep axis; Km=Mean curvature; Kmax=Maximum curvature; Q value=asphericity

Surface	Variable	Mean	SD	Min	Max
	Age	36.8	14	18.0	68
	Male	33			
	Female	65			
	Left eye	49			
	Right eye	49			
Front	K1	42.87	1.29	40.0	46.7
	K2	43.79	1.21	40.6	46.8
	Km	43.32	1.22	40.4	46.8
	Kmax	44.38	1.33	41.0	47.0
	Q value	-0.40	0.15	-1.1	-0.03
Back	K1	-6.08	0.21	-6.8	-5.6
	K2	-6.34	0.22	-6.8	-5.8
	Km	-6.2	0.21	-6.8	-5.7
	Q value	-0.42	0.13	-0.9	-0.2
	Corneal volume	59.3	3.33	48.0	68.7

Anterior corneal characteristics

As seen in Tables 2-3, we found no correlations of the epithelium with the anterior corneal characteristics except in isolated locations. The K1 had a statistically significant but low positive correlation with the central epithelial thickness (p=0.015, r=0.245) and the inner inferior temporal region (p=0.026, r=0.226). The mean curvature (Km; p=0.042, r=0.207), maximum curvature (Kmax; p=0.042, r=0.207), and astigmatism (p=0.217, r=0.127) only correlated with the central epithelium. No relationship was found with K2, Q-value, KP(0), and KP(45).

**Table 2:** Central, superior and nasal data Abbreviations: K1=Flat axis; K2=Steep axis; Km=Mean curvature; Kmax=Maximum curvature; KP(0)=Polar value at 0 degrees; KP(45)=Polar value at 45 degrees; Q value=asphericity

		Central		Inner Superior		Inner Superior Nasal		Inner Nasal		Inner Inferior Nasal	
Surface	Variable	P-Value	R Value	P Value	R Value	P Value	R Value	P Value	R Value	P Value	R Value
Front	K1	0.015	0.245	0.536	-0.064	0.742	0.034	0.423	0.082	0.109	0.164
	K2	0.138	0.152	0.237	-0.121	0.784	-0.028	0.704	0.039	0.541	0.063
	Km	0.036	0.214	0.404	-0.086	0.917	0.011	0.53	0.065	0.233	0.122
	Qvalue	0.203	-0.13	0.896	-0.013	0.144	-0.149	0.146	-0.149	0.619	-0.051
	Kmax	0.042	0.207	0.611	-0.052	0.772	0.03	0.813	0.024	0.271	0.113
	Astigmatism	0.217	0.127	0.21	0.129	0.341	0.098	0.471	0.074	0.402	0.068
	KP(0)	0.775	-0.029	0.938	0.008	0.677	0.043	0.69	0.041	0.93	0.009
	KP(45)	0.739	0.034	0.319	-0.102	0.324	-0.101	0.893	-0.014	0.7	0.04
Back	K1	0.173	-0.14	0.132	0.154	0.599	0.054	0.282	-0.11	0.817	-0.024
	K2	0.764	0.031	0.008	0.267	0.085	0.176	0.518	-0.066	0.373	0.092
	Km	0.585	-0.056	0.04	0.209	0.283	0.11	0.363	-0.093	0.789	0.028
	Qvalue	0.029	0.221	<0.001	0.423	0.011	0.256	0.029	0.221	0.017	0.243
	Corneal Volume	0.281	-0.11	0.593	-0.055	0.398	-0.087	0.947	0.007	0.232	-0.123
	Astigmatism	0.049	-0.202	0.122	-0.159	0.14	-0.152	0.044	-0.206	0.169	-0.142
	KP(0)	0.1	0.169	0.14	0.152	0.221	0.126	0.155	0.146	0.136	0.153
	KP(45)	0.072	0.184	0.89	-0.14	0.817	0.024	0.16	0.145	0.415	0.084

**Table 3:** Inferior and temporal data Abbreviations: K1=Flat axis; K2=Steep axis; Km=Mean curvature; Kmax=Maximum curvature; KP(0)=Polar value at 0 degrees; KP(45)=Polar value at 45 degrees; Q value=asphericity

		Inner Inferior		Inner Inferior Temporal		Inner Temporal		Inner Superior Temporal	
Surface	Variable	P-Value	R Value	P-Value	R Value	P-Value	R Value	P-Value	R Value
Front	K1	0.136	0.152	0.026	0.226	0.155	0.145	0.879	0.016
	K2	0.525	0.065	0.204	0.13	0.562	0.06	0.771	-0.03
	Km	0.252	0.117	0.06	0.192	0.268	0.114	0.99	0.001
	Qvalue	0.524	-0.065	0.59	-0.055	0.573	-0.058	0.608	-0.053
	Kmax	0.36	0.094	0.062	0.19	0.134	0.153	0.749	0.033
	Astigmatism	0.447	0.078	0.129	0.155	0.272	0.113	0.607	0.053
	KP(0)	0.984	0.002	0.996	0.001	0.77	0.03	0.987	0.002
	KP(45)	0.564	0.059	0.976	0.003	0.613	-0.052	0.371	-0.092
Back	K1	0.742	-0.034	0.157	-0.145	0.77	-0.03	0.401	0.086
	K2	0.468	0.075	0.691	0.041	0.279	0.111	0.045	0.204
	Km	0.92	0.01	0.603	-0.053	0.722	0.037	0.146	0.149
	Qvalue	0.055	0.195	0.014	0.25	<0.001	0.347	<0.001	0.404
	Corneal Volume	0.369	-0.092	0.471	-0.074	0.578	-0.057	0.672	-0.044
	Astigmatism	0.212	-0.128	0.020	-0.237	0.115	-0.162	0.177	-0.139
	KP(0)	0.072	0.185	0.378	0.091	0.363	0.094	0.114	0.163
	KP(45)	0.436	0.08	0.445	0.079	0.921	0.01	0.92	-0.01

Posterior corneal characteristics

The Q-value had statistically significant moderately positive correlations with all sectors of the corneal epithelium except the inner inferior sector (p=0.055, r=0.195). The rest of the variables had no statistical significance except for a few isolated locations. The K2 found significance at the inner superior section (p=0.008, r=0.267) and inner superior temporal (p=0.045, r=0.204), the Km at the inner superior sector (p=0.04, r =0.209), and lastly, astigmatism at three locations: central (p=0.049, r=-0.202), inner nasal (p=0.044, r=-0.206), and inner inferior temporal (p=0.02, r=-0.237). The polar values had no significant correlations.

Multivariate analysis

Most variables were insignificant like the monovariate analysis, as seen in Table 4. The posterior surface Q-values had statistically significant effects in various sectors: inner superior (p<0.001), inner inferior nasal (p=0.045), inner inferior temporal (p=0.029), inner temporal (p=0.002), and inner superior temporal (p<0.001).

**Table 4:** Multivariate analysis Abbreviations: K1=Flat axis; K2=Steep axis; Km=Mean curvature; Kmax=Maximum curvature; KP(0)=Polar value at 0 degrees; KP(45)=Polar value at 45 degrees; Q value=asphericity

Surface	Variable	Central	Inner Superior	Inner Superior Nasal	Inner Nasal	Inner Inferior Nasal	Inner Inferior	Inner inferior temporal	Inner Temporal	Inner Superior Temporal
Front	K1	0.315	0.181	0.324	0.417	0.724	0.701	0.441	0.412	0.188
	K2	0.082	0.12	0.167	0.093	0.128	0.167	0.173	0.135	0.087
	Km	0.12	0.142	0.212	0.166	0.29	0.319	0.22	0.202	0.114
	Qvalue	0.329	0.036	0.05	0.083	0.304	0.217	0.367	0.292	0.089
	Kmax	0.312	0.223	0.445	0.534	0.592	0.363	0.152	0.624	0.569
	Astigmatism	0.966	0.643	0.909	0.587	0.416	0.544	0.74	0.869	0.763
	KP(0)	0.495	0.466	0.803	0.974	0.853	0.813	0.648	0.844	0.607
	KP(45)	0.697	0.395	0.441	0.938	0.536	0.493	0.922	0.807	0.548
Back	K1	0.375	0.732	0.681	0.51	0.779	0.881	0.305	0.395	0.326
	K2	0.563	0.12	0.178	0.316	0.361	0.265	0.678	0.3	0.218
	Km	0.942	0.626	0.673	0.83	0.68	0.529	0.812	0.919	0.953
	Qvalue	0.084	<0.001	0.063	0.123	0.045	0.113	0.029	0.002	<0.001
	Corneal Volume	0.455	0.235	0.316	0.482	0.326	0.315	0.389	0.284	0.282
	Astigmatism	0.985	0.363	0.554	0.895	0.788	0.755	0.844	0.508	0.24
	KP(0)	0.145	0.57	0.154	0.183	0.135	0.068	0.363	0.231	0.045
	KP(45)	0.449	0.159	0.467	0.646	0.983	0.935	0.964	0.316	0.141

BAD value

No significant correlations were obtained when looking at the BAD value and the central (r=0.107, p=0.360), inner superior (r=0.062, p=0.581), inner superior nasal (r=0.093, p=0.503), inner nasal (r=0.152, p=0.390), inner inferior nasal (r=0.101, p=0.374), inner inferior (r=0.121, p=0.281), inner inferior temporal (r=0.050, p= 0.552), inner temporal (r=0.017, p=0.582), and inner superior temporal (r=0.130, p=0.325).

## Discussion

It is important to understand the relationship of the epithelium to various corneal characteristics. This can help us determine how the epithelium responds to corneal changes and insults. We know that it is not a static medium and that it reshapes itself to maintain corneal integrity.

Reinstein et al. have suggested that the epithelium serves to maintain corneal uniformity with sudden stromal curvature changes. This has been shown in post-procedure patients like LASIK [8] and corneal cross-linking (CXL) [9]. This tool has also been implicated in keratoconus screening, as there is focal thinning in the area over the cone with a ring of thickening [4,6,10]; this is termed as the “doughnut pattern.” Similarly, this thinning is also seen in post-LASIK ectasia patients [11].

Interestingly, we found no relationship of any factors except moderate correlations with the corneal posterior surface Q-value in almost all sectors. Reinstein et al. examined keratoconus patients and look at the correlation between the steepest keratometry and thinnest epithelium in the middle of the doughnut [6]. They found a strong correlation between the two variables. Therefore, his team theorized that there was more thinning over the cone area.

Our findings suggest there is a possible relationship between the posterior surface of the cornea and the epithelium in normal eyes. The epithelium doesn’t seem to only respond to anterior curvature changes, but its remodeling may begin with initial changes in the posterior cornea. We know that the keratoconus cone begins at the posterior surface of the cornea and then proceeds to the anterior [12-13]. We also know that there are epithelial changes with subclinical and advanced keratoconus eyes [14]. Additionally, there has also been a suggestion that the epithelium may serve as an early prognostic factor in ectatic eyes [11]. Perhaps the moderate correlation amongst the topographic posterior surface parameters may be stronger in eyes with keratoconus or ectasia; this requires further investigation.

Limitations

This study was a single-arm, cross-sectional study in normal eyes with no comparative group. Additionally, patients were selected from a single hospital site, where perhaps a specific subset of the patients present. Lastly, the epithelial maps of the OCT include the tear film in the analysis; therefore, subtle variations may be present due to differences in the corneal exposure time.

## Conclusions

None of the front or back surface parameters had any influence on the epithelial profiles besides the posterior surface Q-value. Variables such as astigmatism or the curvature of the cornea did not correlate. This could perhaps be due to the use of normal corneas with the epithelium intact. Perhaps using stromal curvature values may yield a separate picture. Additionally, the statistically significant, yet clinically insignificant correlation, of the posterior surface Q-value may be enhanced in diseased populations like keratoconus and could indicate epithelial remodeling with early posterior corneal changes.
